# The role of religious leaders and faith organisations in haemoglobinopathies: a review

**DOI:** 10.1186/1471-2326-9-6

**Published:** 2009-08-15

**Authors:** Thelma K Toni-Uebari, Baba PD Inusa

**Affiliations:** 1 Paediatrics Department, West Middlesex University Hospital, Isleworth, Middlesex, UK; 2 Paediatrics Department, Evelina Children's Hospital, Guys and St Thomas Hospital NHS Trust, London, UK

## Abstract

**Background:**

Sickle cell disease (SCD) is now the most common genetic condition in the world including the UK with an estimate of over 12,500 affected people and over 300 new births per year. Blood transfusion therapy plays a very important role as a disease-modifying strategy in severe SCD e.g. primary and secondary stroke prevention and other acute life-threatening complications such as acute chest infections and acute multi-organ failure. Blood transfusion, however, carries a number of risks including alloimmunisation. There is the need to increase the level of awareness and education about SCD and also to increase blood donation drive among affected communities. These communities are mostly ethnic minority populations who are recognised to have poor access to health care services. Due to the strong impact of religion on these populations, faith organisations may provide potential access for health promotion and interventions.

**Methods:**

A literature search was conducted to find studies published between 1990–2008 aimed at examining the influence of religious leaders and faith organisations in health, with particular reference to haemoglobinopathies.

**Results:**

Eleven studies were reviewed covering a variety of health interventions. The findings suggest that involvement of religious leaders and faith organisations in health related interventions improved the level of acceptance, participation and positive health outcomes within the faith communities.

**Conclusion:**

Religious leaders and faith organisations have the potential to influence health education, health promotion and positive health outcomes amongst members of their faith community. They also provide potential access to at-risk populations for increasing awareness about SCD, encouraging health service utilization and ethnic blood donor drives.

## Background

Haemoglobinopathies are a group of inherited disorders of haemoglobin with over 800 recognised types. The two most important clinical syndromes are sickle cell disease and thalassaemia. Thalassaemia is due to reduced production of the affected haemoglobin chains and therefore referred to either as alpha or beta thalassaemia. Beta thalassaemia major is associated with severe anaemia and therefore dependent on blood transfusion, while other forms present a variable degree of severity with anaemia depending on the type of genetic mutation. Sickle cell disease (SCD) on the other hand is due to a single amino acid substitution of the beta chain of haemoglobin that results in the formation of abnormal haemoglobin. Among the most common SCD syndromes are sickle cell anaemia (Homozygous SS), double heterozygote such as sickle haemoglobin C disease (SC) and sickle β thalassaemia disease (Sβ) [1,2].

The World Health Organisation estimates that over 300,000 babies with severe forms of these disorders are born worldwide each year making it the most prevalent inherited disease of mankind [1]. In the UK, SCD is particularly common among people of African and African-Caribbean origin and people whose ancestors come from the Mediterranean region, southern Europe, the Middle East and Asia [2]. There are no UK national registers for this condition but best estimates suggest that there are over 12,500 patients with SCD and about 700–800 patients with thalassaemia [3]. In England, SCD is now the most common genetic condition, affecting more than 1 in 2,000 live births. The birth prevalence in some urban areas may be as high as 1 in 300 [4,5]. Sickle cell disease is now more more common than cystic fibrosis in England, although the distribution of cases is concentrated in London where it is about five times as common. The prevalence is also more in other urban areas [5,6]. About 10 per cent of African-Caribbean people carry the sickle cell trait, whereas 1 in 12 Pakistani individuals carry the thalassaemia trait [7].

The central pathology in SCD is the fact that in deoxygenated states affected haemoglobin form polymers of haemoglobin and 'sickling' distortion leading to occlusion of small blood vessels. It is worth emphasising that both SCD and Thalassaemia undergo haemolysis with anaemia as a common feature. Blood transfusion therapy plays an important role as a disease-modifying strategy for several manifestations of these conditions, including very low haemoglobin, acute chest syndrome, stroke, acute intrahepatic sequestration, acute multi-organ failure syndrome, acute splenic sequestration, aplastic crisis, bacterial and malarial infections and chronic organ failure [8]. Patients may receive simple or chronic blood transfusions episodically or regularly depending on their clinical manifestations. Each transfusion of a unit of red blood cells (RBC) poses a risk of RBC antigen alloimmunisation. This is the development of antibodies to the red blood cells further compromising the success of subsequent blood transfusions. Alloimmunisation, as a result of antigenic discrepancy between patients of African ancestry and predominantly Caucasian blood donors, affects 5 to 50 percent of patients with SCD [9]. Alloimmunisation can be reduced by transfusing phenotypically matched red blood cells [10]. Such units of blood may be found in ethnically matched donors, from directed donors, or by phenotyping donor RBC components [11].

Table 1[12] indicates that the best donor/recipient compatibility for African-Americans is from African-American donors. Data from the UK are not readily available but since SCD affects mostly people from African-Caribbean ancestry and other ethnic minority populations the incidence of alloimmunisation would be reduced by giving patients community matched blood.

**Table 1:** Given 1000 of the indicated donor race, the average probability of at least a 4/6 match for recipients of another race [12].

**Donor Race**	**Recipient Race**				
	**Caucasian**	**African** **American**	**Hispanic**	**Asian** **American**	**Native** **American**
Caucasian	94%	55%	84%	61%	92%
African American	82%	88%	71%	44%	74%
Hispanic	91%	60%	90%	69%	89%
Asian American	76%	45%	69%	93%	79%
Native American	94%	63%	86%	64%	96%

Studies in USA show that although there is a 93 percent probability that RBCs of the E-, C-, Fy (a-), K-, Jk(b-) phenotype would be from an African American donor, only about 10 percent of African Americans in an urban population donate, making it difficult to support the transfusion needs of patients with SCD with these red blood cells alone. On the other hand, although about 90 percent of Caucasian donors in this same setting donate, there is only a 7 percent probability that their RBCs would be of this phenotype [11]. Data from the UK is limited but the pattern may be similar (personal communication), necessitating the need to increase awareness of SCD within the populations at risk and establish a more effective blood donor scheme within these communities. Increased awareness about the services available, such as prenatal diagnosis, will assist at-risk individuals in making informed decisions.

The results of the newborn SCD screening programme in England 2003–2004 demonstrated that the variation in the distribution of different ethnic minority populations across the country was mirrored by the sickle cell carrier distribution [6]. Providing culturally-sensitive health information and health education to people from ethnic minorities has been a challenge due to factors which include communication difficulties and poor access to health services [13]. Religion is traditionally believed to be particularly salient to people from groups that are likely to be most affected by sickle cell disorders and thalassaemia major [14]. A potential avenue for disseminating information and increasing awareness about SCD is through the influence of religious leaders and faith organisations. Increasingly many faith organisations are being used to deliver preventive health programmes.

This review aims to examine whether religious leaders and faith organisations have the potential to influence health outcomes and health services utilization with particular reference to haemoglobinopathies. Religious leaders in this regard refers to ministers of various religions within the context of organised religiosity including priests, bishops, imams, monks etc, authoritative religious figures and scholars with capacity to influence religious practice.

## Methods

### Search strategy

Search for relevant studies were carried out using MEDLINE, Cochrane Library Database, PsychInfo and CINAHL (Cumulative Index to Nursing & Allied Health Literature). Database searches were supplemented by manual searches of relevant journals, tracking of citations from citation lists of relevant publications, and searches of Sickle cell Society webpage and other related websites using Google search engine. Key search words included religion, religious leaders, priests, imams, pastors, sickle cell disease, haemoglobinopathies, thalassaemia, faith organisations, health, influence, role, interventions etc. in various combinations.

### Inclusion criteria

Although the influence of religion on health has been on for many years databases were searched for studies from 1990 till present (2008) mainly to provide more current and relevant information. Studies were included if they reported the influence of religious leaders in health-related outcomes or if they involved faith organisations in the administration of interventions possibly in collaboration with health personnel, if the effects of the intervention were documented and if the interventions were targeted at haemoglobinopathies. Studies involving other health-related interventions were to be included if too few studies were obtained for haemoglobinopathies. Only studies published in English language were included.

### Study selection

All the citations obtained from the electronic databases and other searches were screened by their titles and abstracts to identify potentially relevant citations. All the studies identified at this stage were selected and full text copies were obtained. Further scrutiny of the full text documents was carried out and studies that met the inclusion criteria were selected for the review after excluding duplication.

## Results

Of the eleven studies reviewed four were qualitative studies, four descriptive studies, and three randomised clinical trials. The interventions were heterogeneous with four of the studies involving haemoglobinopathies, three health promotion services, and one each for smoking cessation, improving vaccination uptake, mammography promotion and use of contraception. Most of the studies (6) were carried out in the USA, two in Pakistan, one in the UK, one in Thailand and one Saudi Arabia. The findings have been categorised below based on the broad objectives of the review.

### Studies that examine the role of religious leaders

In a pilot survey, Ahmed *et al*. [15] reported the effect of consulting renowned Islamic scholars before the introduction of a service for prenatal diagnosis of β-thalassaemia in Pakistan. The Islamic scholars ruled that a pregnancy could be terminated if the foetus was affected by a serious genetic disorder and if termination was before 120 days (17 weeks) of gestation. Information about this ruling was widely circulated to affected families via newspaper and TV adverts and information booklets including local language translations by a charity organisation. 141 randomly selected couples with an affected child were interviewed one year after the start of the service. 72% knew of the availability of prenatal diagnosis. Of the 60 couples that had a viable pregnancy, thirty-two were aware about prenatal diagnosis but only 18 (56%) used the service. The main reasons for non-utilization of prenatal diagnosis were the cost of the test and fear of undergoing the test, though some gave no clear explanation. 102 couples (91% of the couples who had not completed their families) were in favour of prenatal diagnosis in subsequent pregnancies. 73.5% would request it unconditionally while 26.5% would request it if it was free of cost. The authors concluded that prenatal diagnosis was feasible and acceptable in a Muslim country such as Pakistan.

In another qualitative study Alkuraya and Kilani [16] examined the attitude of Saudi families affected with haemoglobinopathies towards prenatal diagnosis and abortion and evaluated the effect of education on the religious ruling (Fatwa) on such attitudes. The Fatwa implies that abortion is permissible if a diseased foetus is diagnosed in the first 120 days. 32 Muslim families with children affected by Sickle cell anaemia and/or Thalassaemia were interviewed using a pre-structured questionnaire. Most of the participants accepted prenatal diagnosis (81.3%) but the attitude towards abortion was greatly affected by religious values. Education about the religious ruling significantly affected parents' attitude towards accepting abortion and prenatal diagnosis. The authors concluded that religious education as an intervention played a positive role on parental attitude towards prenatal diagnosis and abortion.

Swaddiwudhipong *et al *[17] evaluated the effects of an intervention by monks to change smoking behaviour and attitudes in a rural community in Thailand in March 1991. The survey group consisted of 372 individuals in the intervention and 664 in the control groups. Many former smokers (80.3%) in the intervention village cited the suggestion of a monk as one important reason for quitting compared with 25.6% in the control village (P = 0.000). Other findings in outcome measures led the authors to conclude that religious leaders may be helpful in a community-based smoking prevention programme. This study is fraught with poor methodological issues and confounding factors which the authors have not addressed either by study design or by statistical analysis. The use of self-reporting and the absence of blinding increase potential for bias which could affect the validity of the results.

### Studies that examine the role of faith organisations

In a recently published US study Price *et al *[18] report the impact of Sickle Cell Sabbath on blood donation in St Louis. Sickle Cell Sabbath is a community educational outreach programme designed to increase awareness about SCD and promote blood donation within the African-American faith community for children with SCD. Thirteen African-American churches participated in the programme and sponsored 34 blood drives. The findings indicated that the percentage of first-time blood donors at Sabbath blood drives was 60% (428 of 699) compared with 12.2% (21,516 of 175,818) for the entire St. Louis Metropolitan area (p = 0.001). The authors concluded that an educational program that engaged the African-American faith community more than quadrupled the rate of first-time blood donors when compared to the general community over the four-year period.

Yanek *et al *[19] reported a clinical trial to test the impact on the cardiovascular risk profiles of African American women aged 40 years and older after participation in one of three church-based nutrition and physical activity interventions. 529 women from 16 churches participated in the study. One year after the program initiation the intervention participants were found to achieve clinically important improvements in their cardiovascular disease risk profiles, which did not occur in the control group. The authors concluded that church-based interventions could significantly benefit the cardiovascular health of African American women. The problems encountered with the randomisation process, recruitment of participants, absence of blinding, problems with implementation of the standard intervention and high loss to follow-up particularly with the control group (60%) increase the potential for bias and the influence of confounding factors.

In another US-based randomized control study Daniels *et al *[20] compared the effect of an on-site adult vaccination programme involving 15 churches (intervention group) and a control comparison group. The study sample consisted of elderly people aged >65 years (N = 186). The intervention faith-based group were found to be significantly more likely to receive influenza vaccinations and pneumococcal vaccination than the comparison group (P < 0.001). The authors concluded that the faith-based organisation adult vaccination programme was effective in increasing vaccination rates. The role of confounding factors was not clearly stated and the potential of community support within the religious may have influenced the outcome.

Duan *et al *[21] reported a randomized trial in Los Angeles to study the delivery of mammography promotion through an ethnically diverse group of churches from 1996 to 1998. 1443 women aged 50–80 years were recruited from 30 churches for the study, randomised into intervention and control groups for a 2-year intervention incorporating peer telephone counselling sessions. The primary outcome measure was annual mammography screening adherence status. By the end of one year the intervention group showed statistically significant difference in output measure (p = 0.029). The authors concluded that partnerships between the public health and faith communities were potentially effective conduits to promote maintenance of widely endorsed health behaviours such as regular cancer screening. Although the process of randomisation was not described in this publication logistic regression analysis was used to identify matching variables. Blinding was not specifically reported but the use of peer counsellors to collect the one year data could potentially affect their blind status to the intervention and introduce bias, as could reliance on self-report data for mammography use by participants.

### Studies that examine the perception of the role of religious leaders by their members

In a UK-based qualitative study Ahmed *et al *[22] explored the influence of faith and religion and the role of community leaders in prenatal decision-making for sickle cell disorders and thalassaemia major. Participants for the study, aged between 18 and 45 years, were drawn from four 'faith' communities *viz *Pakistani Muslim, Indian Hindu, Indian Sikh and African-Caribbean Christian, selected because of the high prevalence of either sickle cell disorders or thalassaemia major within them. Using eight focus groups the similarities and differences between the groups due to faith or religious beliefs were explored. Prenatal screening was considered uncontroversial and did not require religion or faith in making decisions. However, prenatal diagnosis and the option of termination of pregnancy presented a dilemma. All the faith groups generally agreed that while they may explore their religion's stance on the issue it would not influence their decision about termination of pregnancy and individuals would have to make their own decision, giving precedence to their own moral beliefs and values. Religious leaders were considered not to have an important role to play on a one-to-one level with people on personal reproductive issues because they were more likely to provide biased opinions than advice based on medical knowledge, and they were unlikely to be aware of the severity of the medical conditions and of their impact on the affected child and family.

The small sample size for this study and the absence of random selection of the participants are possible sources of bias as they may not be representative of the general population and these could affect the generalisability of the findings.

Ali and Ushijima [23] explored the perception of adult males in Pakistan regarding the influence of the 'religious factor' in their use of modern contraceptive methods, and their views on the role of religious leaders in community education. 180 married male adults participated in the cross-sectional survey. Respondents suggested that the involvement of religious leaders in reproductive health programmes was essential for the programmes' effectiveness in rural areas. They were of the opinion that religious leaders could contribute positively to community education. The study concluded that involving religious leaders in rural settings could enable reproductive health programmes and services to reach more conservative groups in society.

### Studies that examined the perception of religious leaders to delivering health promotion interventions

Cantanzaro [24] reported a cross-sectional survey of a national multidenominational sample of 349 pastors representing over 80 Christian denominations. The study compared the perceptions of Pastors with and without organised Congregational Health Ministries (CHMs)- a movement that organises church-based health services under the coordination of a health personnel, mainly health promotion, disease prevention and support services. The authors found that pastors with CHMs were significantly more involved in health promotion and disease prevention activities. Pastors without CHMs perceived a need for congregations to be involved in health-related services and were willing to become involved if resources were available. The authors concluded that because of the long-term trusting relationships existing between congregants and their leaders, religious congregations would be ideal for providing cost-effective community-based health promotion and disease prevention services.

In another US-based study Markens *et al *[25] assessed pastor-level factors that affect the implementation of community-based health promotion programmes in black churches in Los Angeles. The views of 16 pastors of black churches were obtained using semi-structured interviews. Their findings suggested that although Black pastors were willing to participate and appreciated being included in health research, minorities' history of being underserved and exploited could lead to suspicion and reluctance to participate in such programmes.

## Discussion

The number of patients diagnosed with sickle cell disease in the UK is increasing making it of public health relevance. Immigration is changing the demography with more adults immigrating to the UK from Africa and the Caribbean, some with sickle carrier status or with established SCD.

The NHS has shown increasing commitment to the diagnosis and management of patients with SCD over the recent years with the establishment of routine screening for sickle cell and thalassaemia and provision of guidelines for the management of sickle cell patients. However the full benefits of these services depend on their utilisation.

The majority of those affected by SCD are from the ethnic minority population. There is a large evidence base to show that disparities exist in access to healthcare services for ethnic minority populations in the UK [13], highlighting the need for a culturally appropriate means of health education and health promotion.

Religion plays a prominent role in the social and cultural life of individuals from the ethnic minority population with people identifying with a variety of faith-based groups where their leaders have a considerable measure of influence built on a combination of trust and culture. Whilst not being the only outreach medium for health education the faith community is characterised by a large population of people who show commitment, effective social and community support, socio-cultural belief systems acceptable to the groups and some element of trust in their leaders. Thus religious leaders provide potential access to this underserved ethnic minority population for health promotion, health education and improvement in utilization of health services.

The findings from the studies in this review indicate a positive outcome for the health interventions delivered through faith organisations irrespective of the mode of delivery of the intervention, whether through collaboration with health care establishments or through trained lay members of the religious community. The success of these interventions may have little to do with specific religions but more to do with the existing and effective social support system existing within faith communities and the fact that they are more likely to commit to healthier lifestyle changes or interventions. These attributes increase the chance of success for health interventions such as vaccination programmes. They may also introduce bias; thereby confounding factors during evaluation of the effects of such interventions as observed in the study by Yanek et al [19].

Some of the studies demonstrated that involvement of religious leaders were influential in encouraging the utilization of available health services, albeit indirectly. The involvement of influential Islamic scholars and their legislation on the upper limit of permissibility of pregnancy termination enhanced the utilization of health services in Pakistan and Saudi Arabia. [15,16]. In the authors' experience individuals who were hesitant in using prenatal diagnosis were relieved to learn that Islam permitted termination of pregnancy under special circumstances and this had resulted in increased utilization of the services. Involvement and enlightenment of religious leaders on health interventions have the potential of encouraging the use of these services among their members especially if they are aware of the endorsement by their leaders.

The success of health-related interventions correlates directly with the attitude and commitment of the religious leaders to the project. Most of the studies indicated that religious leaders were willing to be involved in health related projects however some leaders may be hampered by very busy schedules, limited resources or even a distrust or suspicion of the health system or its intensions towards members of their congregation [25]. These challenges would need to be taken into consideration to ensure favourable outcomes for future interventions.

The role of religious leaders as perceived by the followers was highlighted. Their involvement in general and reproductive health programmes was perceived to be essential for the programmes' effectiveness [23]. In a telephone survey to assess barriers and motivators to blood and cord blood donation in young African-American women 17% of the participants would donate blood if encouraged by their religious leaders [26].

However, on crucial personal issues such as decisions-making about prenatal diagnosis and abortion people tended to defer to their individual faith, morals and information from relevant health personnel before making their decisions [22]. Religious leaders were perceived to be relatively uninformed about their situations from a health or scientific perspective. Religious representatives however thought otherwise, emphasizing that their role was to support people rather than tell them what to do [27].

The findings of this review are consistent with the review on effectiveness of health programmes in faith based organisations by De Haven *et al *[28]. Unlike this review, however, the studies were restricted to faith based organisations in the United States and were all within Christian settings. The similarity of the findings indicates that the positive health outcomes of faith based organisations are not limited by religion or geographical location.

One of the areas to explore the potential influence of religious leaders is in helping parents of newly-diagnosed SCD patients to cope with and accept the fact of the diagnosis. Many parents tend to go through a period of denial which could last from a few weeks to many years and this could potentially delay the commencement and use of antibiotic prophylaxis for their children putting them at risk for infections and other consequences.

The access provided by faith organisations for health education cannot be over-emphasised. Genetic counselling provided within a culturally sensitive environment would be helpful especially since there is a high proportion of individuals with carrier status for sickle cell disease (1 in 10) and some of them may have moral and religious reasons for not considering prenatal diagnosis or termination of pregnancy [29]. The consensus of the respondents in the aforementioned study was that SCD was best controlled through effective genetic counselling rather than through prenatal diagnosis and selective abortion of the affected pregnancies.

The potential for educating communities at risk for SCD about the condition and the role of blood transfusion in the management of haemoglobinopathies is one of the major considerations for this review. Altruism and the awareness of the need for blood are major motivators to donate blood [30]. Although data from the UK are not readily available communication with relevant staff of the National Blood Service at Tooting, London has confirmed that the proportion of blood donors from African-Caribbean origin was very low compared with Caucasians. Given the potential risk for alloimmunisation in the absence of phenotypically matched red blood cells there is need for establishment of ethnic blood donation drives. The success of The Sickle Cell Sabbath and the Charles Drew Blood donation programmes confirm this stance [18,31]. Grassineau *et al *[32] reported the utilization of an anthropologic approach and cultural mediation to improve minority blood donation within a migrant community. There are in existence some local or regional blood donor drives in the UK. It is our belief that involvement of religious leaders and faith organisations would extend the reach and outcomes of such interventions.

Another potential benefit of the combination of education about SCD and the influence of religious leaders is combating the issue of stigmatization to which patients with SCD and their families are subjected.

Religious leaders are a very heterogeneous group with variations in levels of education and medical knowledge, cultural beliefs and practices, acceptance of science or perceived 'western' or modern practices, biases, variations in interpretation of their holy scripts etc. They share some of the concepts and beliefs about disease causation and inhibitions held by their members e.g. views on blood donation or transfusion. Thus there will be the need for education, enlightenment and sharing of information with religious leaders using culturally-sensitive and appropriate media in order to channel their influence on their members towards positive outcomes for health.

An important observation from this review is the paucity of published studies from the UK on sickle cell disease, haemoglobinopathies, blood compatibility issues and the role of religious organisations or religious leaders on health-related matters. There is need for more research to be carried out in the UK to add to body of evidence available locally and for purposes of comparison.

### Limitations

There were limitations to this review. Some of the studies available were of poor methodological quality, small sample size and designs that do not provide strong quality of evidence. Publication bias cannot be ruled out considering that most of the reviewed data were drawn from published studies. Other potential sources of bias include restricting the review to studies published in English language thus missing out on any relevant studies published in other languages. The authors acknowledge that we may not have exhausted all the publications relevant to this review but we are of the view that the findings from the studies not included may not differ significantly from the present results.

In spite of these limitations the findings of the review has shed some light in answering the research question about the influence of religious leaders and faith organisations in haemoglobinopathies.

## Conclusion

SCD is now the most common genetic condition in the UK and presents a public health challenge. Whilst improvement in health services for patients with SCD has enhanced overall management and quality of life and survival; there are still challenges for health access for many of the at-risk populations. Greater awareness and education about SCD and blood donation is necessary for these populations to encourage greater utilization of the available health services for SCD patients.

The findings from the review indicate that religious leaders and religious organisations have influence over their followers or congregations and provide potential access to these underserved, at-risk populations for increasing awareness, encouraging health service utilization and designing ethnic blood donor drives.

## Competing interests

The authors declare that they have no competing interests.

## Authors' contributions

TKT conducted the literature search, carried out the review and drafted the manuscript. BI conceived of the study and participated in the review and coordination. Both the authors read and approved the final manuscript.

## Pre-publication history

The pre-publication history for this paper can be accessed here:



## References

[B1] WHO Sickle-cell disease and other haemoglobin disorders, Fact sheet N°308. http://www.who.int/mediacentre/factsheets/fs308/en/.

[B2] Anie KA, Steptoe A, Ball S, Dick M, Smalling BM (2002). Coping and health service utilisation in a UK study of paediatric sickle cell pain. Arch Dis Child.

[B3] Lucas SB, Mason DG, Mason M, Weyman D (2008). A Sickle Crisis? A report of the National Confidential Enquiry into Patient Outcome and Death. London.

[B4] Sickle cell disease in childhood: Standards and guidelines for clinical care, NHS Sickle Cell and Thalassaemia Screening Programme. http://sct.screening.nhs.uk/cms.php?folder=2493#fileid10762.

[B5] Streetly A, Latinovic R, Hall K, Henthorn J (2009). Implementation of universal newborn bloodspot screening for sickle cell disease and other clinically significant haemoglobinopathies in England: screening results for 2005–7. Journal of Clinical Pathology.

[B6] Streetly A, Clarke M, Downing M, Farrar L, Foo Y, Hall K, Kemp H, Newbold J, Walsh P, Yates J, Henthorn J (2008). Implementation of the universal newborn screening programme for sickle cell disease in England: results for 2003–2005. J Med Screen.

[B7] Modell B, Anionwu A (1996). Guidelines for screening for haemoglobin disorders: service specifications for low- and high-prevalence DHAs. Ethnicity and health: reviews of literature and guidance for purchasers in the areas of cardiovascular disease, mental health and haemoglobinopathies.

[B8] Shulman IA (2006). Prophylactic phenotype matching of donors for the transfusion of nonalloimmunized patients with sickle cell disease. Immunohematology.

[B9] Rogers ZR (2006). Review: clinical transfusion management in sickle cell disease. Immunohematology.

[B10] Castro O, Sandler SG, Houston-Yu P, Rana S (2002). Predicting the effect of transfusing only phenotype-matched RBCs to patents with sickle cell disease: theoretical and practical implications. Transfusion.

[B11] Flickinger C (2006). In search of red blood cells for alloimmunized patients with sickle cell disease. Immunohematology.

[B12] Beatty PG, Boucher KM, Mori M, Milford EL (2000). Probability of Finding HLA-mismatched Related or Unrelated Marrow or Cord Blood Donors. Human Immunology.

[B13] Szczepura A (2005). Access to health care for ethnic minority populations. Postgrad Med J.

[B14] Anionwu EN, Atkin K (2001). The Politics of Sickle Cell and Thalassaemia.

[B15] Ahmed S, Saleem M, Sultana N, Raashid Y, Waqar A, Anwar M, Modell B, Karamat KA, Petrou M (2000). Prenatal Diagnosis of Beta-Thalassaemia in Pakistan: Experience in a Muslim Country. Prenatal Diagnosis.

[B16] Alkuraya FS, Kilani RA (2001). Attitude of Saudi Families Affected with Hemoglobinopathies towards Prenatal Screening and Abortion and the Influence of Religious Ruling (Fatwa). Prenatal Diagnosis.

[B17] Swaddiwudhipong W, Chaovakiratipong C, Nguntra P, Khumklam P, Silarug N (1993). A Thai monk: An agent for smoking reduction in a rural population. Int J Epidemiol.

[B18] Price CL, Johnson MT, Lindsay T, Dalton D, Watkins AR, DeBaun MR (2009). The Sickle Cell Sabbath: A Community Program Increases First-time Blood Donors in the African-American Faith Community. Transfusion.

[B19] Yanek LR, Becker DM, Moy TF, Gittelsohn J, Koffman DM (2001). Project Joy: Faith based cardiovascular health promotion for African American women. Public health reports.

[B20] Daniels NA, Juarbe T, Moreno-John G, Pérez-Stable EJ (2007). Effectiveness of adult vaccination programs in faith-based organizations. Ethnicity & disease.

[B21] Duan N, Fox S, Derose KP, Carson S, Stockdale S (2005). Identifying Churches for Community-Based Mammography Promotion: Lessons From the LAMP Study. Health Educ Behav.

[B22] Ahmed S, Atkin K, Hewison J, Green JM (2006). The influence of faith and religion, and the role of religious and community leaders in prenatal decisions for sickle cell disorders and thalassaemia major. Prenatal Diagnosis.

[B23] Ali M, Ushijima H (2005). Perceptions of men on role of religious leaders in reproductive health issues in rural Pakistan. Journal of Biosocial Science.

[B24] Cantanzaro AMM, Meador KG, Koenig HG, Kuchibhatla M, Clipp EC (2007). Congregational Health Ministries: A National Study of Pastors' Views. Public Health Nursing.

[B25] Markens S, Fox SA, Taub B, Gilbert ML (2002). Role of Black churches in health promotion programs: lessons from the Los Angeles Mammography Promotion in Churches Program. American Journal of Public Health.

[B26] Grossman B, Watkins AR, Fleming F, DeBaun MR (2005). Barriers and motivators to blood and cord blood donation in young African-American women. Am J Hematology.

[B27] Atkin K, Ahmed S, Green J, Hewison J (2008). Decision making and ante-natal screening for sickle cell and thalassaemia disorders: to what extent do faith and religious identity mediate choice?. Current Sociology.

[B28] DeHaven MJ, Hunter IB, Wilder L, Walton JW, Berry J (2004). Health programs in faith-based organizations: Are they effective?. American Journal of Public Health.

[B29] Durosinmi MA, Odebiyi AI, Adediran IA, Akinola NO, Adegorioye DE, Okunade MA (1995). Acceptability of Prenatal Diagnosis of Sickle Cell Anaemia (SCA) by Female Patients and Parents of SCA Patients in Nigeria. Social Science and Medicine.

[B30] Glynn SA, Kleinman SH, Schreiber GB, Zuck T, McCombs S, Bethel J, Garratty G, Williams AE (2002). Motivations to donate blood: demographic comparisons. Retrovirus Epidemiology Donor Study. Transfusion.

[B31] Isaak EJ, LeChien B, Lindsey T, Debaun MR (2006). The Charles Drew program in Missouri: a description of a partnership among a blood center and several hospitals to address the care of patients with sickle cell disease. Immunohematology.

[B32] Grassineau DPK, Ducourneau A, Duboz P, Boëtsch G, Chiaroni J (2007). Improving minority blood donation: anthropologic approach in a migrant community. Transfusion.

